# Application of the Ribosomal DNA ITS2 Region of *Physalis* (Solanaceae): DNA Barcoding and Phylogenetic Study

**DOI:** 10.3389/fpls.2016.01047

**Published:** 2016-07-19

**Authors:** Shangguo Feng, Mengying Jiang, Yujun Shi, Kaili Jiao, Chenjia Shen, Jiangjie Lu, Qicai Ying, Huizhong Wang

**Affiliations:** ^1^Zhejiang Provincial Key Laboratory for Genetic Improvement and Quality Control of Medicinal Plants, College of Life and Environmental Sciences, Hangzhou Normal UniversityHangzhou, China; ^2^School of Foreign Languages, Zhejiang Gongshang UniversityHangzhou, China

**Keywords:** *Physalis*, molecular identification, DNA barcoding, phylogenetic relationship, ITS2

## Abstract

Recently, commercial interest in *Physalis* species has grown worldwide due to their high nutritional value, edible fruit, and potential medicinal properties. However, many *Physalis* species have similar shapes and are easily confused, and consequently the phylogenetic relationships between *Physalis* species are poorly understood. This hinders their safe utilization and genetic resource conservation. In this study, the nuclear ribosomal ITS2 region was used to identify species and phylogenetically examine *Physalis*. Eighty-six ITS2 regions from 45 *Physalis* species were analyzed. The ITS2 sequences were aligned using Clustal W and genetic distances were calculated using MEGA V6.0. The results showed that ITS2 regions have significant intra- and inter-specific divergences, obvious barcoding gaps, and higher species discrimination rates (82.2% for both the BLASTA1 and nearest distance methods). In addition, the secondary structure of ITS2 provided another way to differentiate species. Cluster analysis based on ITS2 regions largely concurred with the relationships among *Physalis* species established by many previous molecular analyses, and showed that most sections of *Physalis* appear to be polyphyletic. Our results demonstrated that ITS2 can be used as an efficient and powerful marker in the identification and phylogenetic study of *Physalis* species. The technique provides a scientific basis for the conservation of *Physalis* plants and for utilization of resources.

## Introduction

*Physalis* L., one of the most important genera in the family *Solanaceae*, contains 75–120 species, which are mainly distributed in tropical, and temperate regions of America, although there are a few species in Eurasia and Southeast Asia (Chinese academy of sciences, 1978; Martinez, 1998; Maggie, 2005; Wei et al., 2012; Zamora-Tavares et al., 2015). There are five *Physalis* species and two varieties in China and they are mostly found in the east, central, south, and southwest regions of China (Chinese academy of sciences, 1978). They are rich in vitamins, minerals, and antioxidants, and have potential medicinal properties, including anti-bacteria, anti-inflammatory, and anti-cancer actions (Ji et al., 2012; Wei et al., 2012; Hong et al., 2015). Many *Physalis* species are horticulturally and economically important, and commercial interest has increased in many regions of the world over recent decades (Wu et al., 2006; Wei et al., 2012; Ding et al., 2014). Some *Physalis* species, including *Physalis alkekengi, Physalis pubescens, Physalis peruviana*, and *Physalis philadelphica* have been extensively cultivated for their edible fruit, medicinal properties, or as ornamental plants in many regions of the world, such as China and Mexico (Wei et al., 2012; Zamora-Tavares et al., 2015). Currently, however, most natural *Physalis* species are rare due to habitat destruction and increased urbanization.

**Figure 1:**
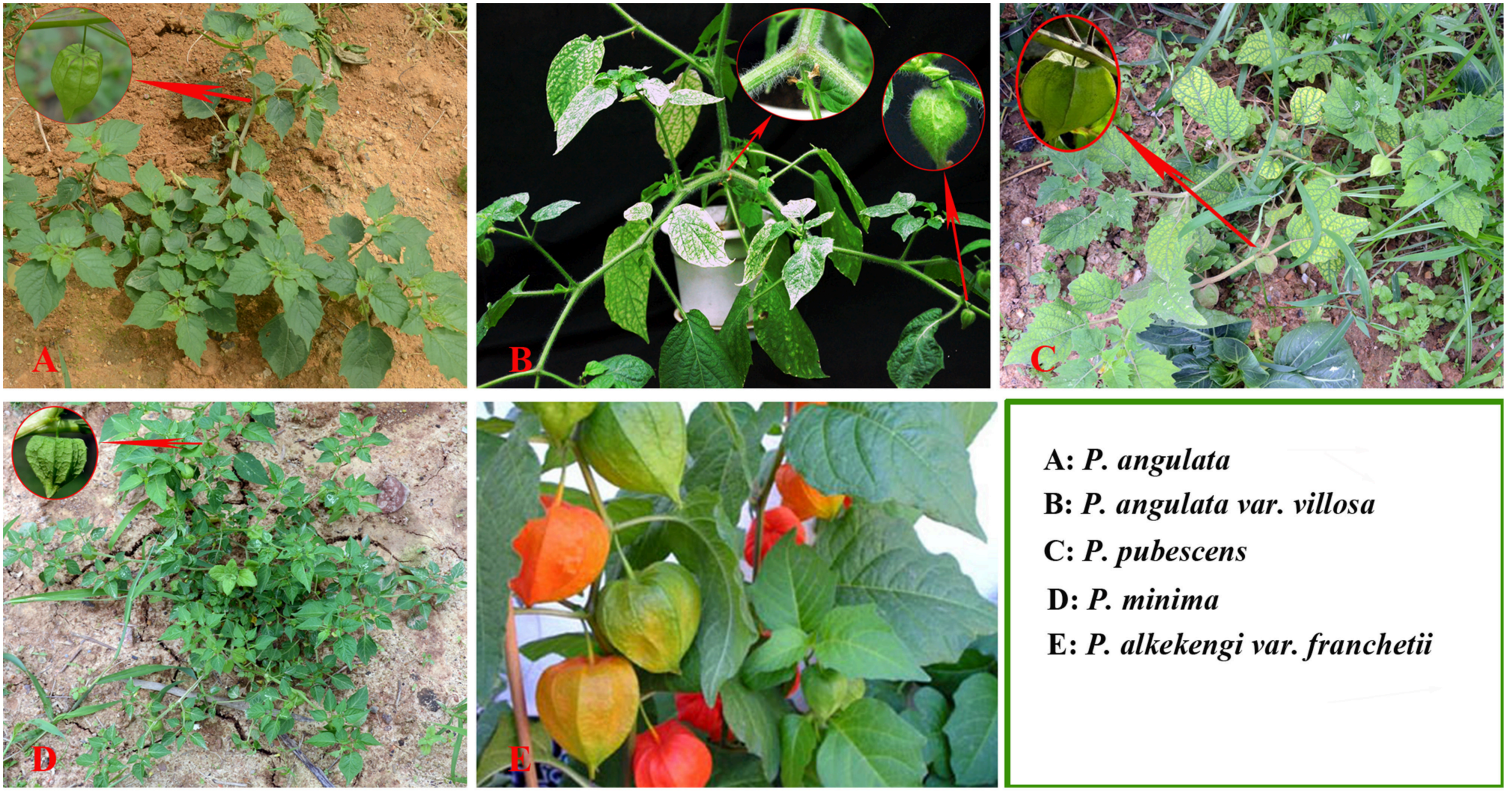
**Plant morphology of ***Physalis*** species (A, ***P. angulate***; B, ***P. angulata*** var. ***villosa***; C, ***P. pubescens***; D, ***P. minima***; E, ***P. alkekengi*** var. ***franchetii***) in their natural habitats**.

The accurate identification of *Physalis* species is extremely important in *Physalis* plant breeding programs and for the conservation of genetic resources. Traditionally, identification of *Physalis* species has been dependent on morphological characteristics (Menzel, 1951; Axelius, 1996; Martinez, 1998; Vargas et al., 2001). Phenotypic characteristics, however, are often affected by plant variability and growth habitats (Maggie, 2005; Vargas-Ponce et al., 2011; Wei et al., 2012). In addition, plants of the genus *Physalis* have a similar shape and their morphological characteristics are easily confused (Figure 1). Molecular markers are independent of environmental conditions and have now emerged as important tools for modern taxonomists (Feng et al., 2014). Some DNA marker systems, including simple sequence repeat (SSR), and inter-simple sequence repeats (ISSR), have been used to genetically study *Physalis* plants (Vargas-Ponce et al., 2011; Wei et al., 2012; Zamora-Tavares et al., 2015). In addition, the DNA sequences of a few genes, including the internal transcribed spacer (ITS) of the nrDNA, the *Waxy* gene, and chloroplast regions (*ndh*F and *trnL*F), have also been used to assess the phylogeny of *Physalis* and their relationship to other genera in the Solanaceae family (Maggie, 2005; Olmstead et al., 2008).

DNA barcoding is a relatively new taxonomic method that uses short DNA sequences of standard genome regions to make fast, efficient, and reliable species identifications (Hebert et al., 2004; Chen et al., 2010; Gao et al., 2010a; Hajiahmadi et al., 2013; Feng et al., 2015). As an efficient tool for species identification, DNA barcoding has become important in biological systematics, and identification (Chen et al., 2010; Gao et al., 2010a; Liu et al., 2012; Dong et al., 2015; Feng et al., 2015; Wang et al., 2015). Recently, several regions, including *mat*K, *rbc*L, *psb*A–*trn*H, *atp*F–*atp*H, *ycf* 1, and ITS, have been advocated as potential standard DNA barcodes for different taxonomic groups in plants (Chase et al., 2005; Kress et al., 2005; Kress and Erickson, 2007; Lahaye et al., 2008a; Cbol Plant Working Group, 2009; Yao et al., 2009; Parveen et al., 2012; Dong et al., 2015; Larranaga and Hormaza, 2015). ITS2, a sub-region of the nuclear ribosomal ITS, has also been proposed as a novel universal DNA barcode to identify herbs based on 6600 samples of 4800 species (Chen et al., 2010). Compared with whole ITS region, ITS2 was more suitable for species identification because of its short length, and high efficiency for PCR amplification (Chen et al., 2010; Gao et al., 2010a; Han et al., 2013). In addition, the secondary structures of ITS2 sequences could be used as molecular morphological characteristics for species identification (Grajales et al., 2007; Yao et al., 2010). It has been proposed that the ITS2 sub-region should be the standard molecular marker for species authentication and for plant phylogenetic analysis (Schultz and Wolf, 2009; Chen et al., 2010; Gao et al., 2010a,b; Yao et al., 2010; Pang et al., 2011; Liu et al., 2012; Gu et al., 2013; Marghali et al., 2015; Zhao et al., 2015).

In this study, we used ITS2 region to barcode *Physalis* and then applied it as a phylogenetic marker to infer the genetic relationships among *Physalis* species.

## Materials and methods

### Plant materials

In total, 86 samples of 45 species from the genus *Physalis* were collected in this study (Table 1). Thirty-one specimens of five species sampled from the main distribution areas in China were used for sequencing (Table 1). Other published *Physalis* ITS2 sequences were downloaded from GenBank (Clark et al., 2016). The species were verified and confirmed using the specimens stored in the herbarium at the Institute of Botany, Chinese Academy of Sciences, Beijing, China (http://www.nhpe.org). All corresponding voucher samples were deposited in the Zhejiang Provincial Key Laboratory for Genetic Improvement and Quality Control of Medicinal Plants, Hangzhou Normal University.

**Table 1:** **Voucher information and GenBank accession numbers for ***Physalis*** plant samples and ***Nicandra physaloides*** (Outgroup) in this study**.

**Subgenus**	**Section**	**Species name**	**Voucher No**.	**Locality information**	**GenBank Accession No**.
*Rydbergis*	*Angulatae*	*P. angulata* L.	PHZ0001	Xiaoshan, Hangzhou, Zhejiang, China	KX147482
		*P. angulata* L.	PHZ0002	Lin'an, Hangzhou, Zhejaing, China	KX147483
		*P. angulata* L.	PHZ0003	Pujiang, Jinhua, Zhejiang, China	KX147484
		*P. angulata* L.	PHZ0004	Yueqing, Wenzhou, Zhejiang, China	KX147485
		*P. angulata* L.	PHZ0005	Luotian, Huanggang, Hubei, China	KX147486
		*P. angulata* L.	PHZ0006	Xiajin, Dezhou, Shandong, China	KX147487
		*P. angulata* L.	PHZ0007	Baohua, Honghe, Yunnan, China	KX147488
		*P. angulata* var. *villosa* Bonati in Gagn.	PHZ1001	Linhai, Taizhou, Zhejiang, China	KX147489
		*P. angulata* var. *villosa* Bonati in Gagn.	PHZ1002	Linhai, Taizhou, Zhejiang, China	KX147490
		*P. angulata* var. *villosa* Bonati in Gagn.	PHZ1003	Changqian, Hangzhou, Zhejiang, China	KX147491
		*P. angulata* var. *villosa* Bonati in Gagn.	PHZ1004	Yiwu, Jinhua, Zhejiang, China	KX147492
		*P. acutifolia* (Miers) Sandwith	–	GenBank	AY665876
		*P. crassifolia* Benth.	–	GenBank	AY665889
		*P. crassifolia* Benth.	–	GenBank	AY665890
		*P. lagascae* Roem. and Schult.	–	GenBank	AY665898
		*P. microcarpa* Urb. and Eckman	–	GenBank	AY665903
		*P. philadelphica* Lam.	–	GenBank	AY665871
	*Campanulae*	*P. campanulata* Standl. and Steyerm	–	GenBank	AY665882
		*P. glutinosa* Schlecht.	–	GenBank	AY665892
	*Carpenterianae*	*P. carpenteri* Riddell	–	GenBank	AY665851
		*P. carpenteri* Riddell	–	GenBank	AY665852
	*Coztomatae*	*P. chenipodifolia* Lam.	–	GenBank	AY665883
		*P. coztomatl* Dunal	–	GenBank	AY665888
		*P. coztomatl* Dunal	–	GenBank	AY665887
		*P. greenmanii* Waterf.	–	GenBank	AY665893
		*P. hintonii* Waterf.	–	GenBank	AY665895
		*P. hintonii* Waterf.	–	GenBank	AY665896
	*Epeteiorhiza*	*P. pubescens* L.	PHZ2001	Faku, Shenyang, Liaoning, China	KX147493
		*P. pubescens* L.	PHZ2002	Guta, Jinzhou, Liaoning, China	KX147494
		*P. pubescens* L.	PHZ2003	Changhai, Dalian, Liaoning, China	KX147495
		*P. pubescens* L.	PHZ2004	Chaoyang, Zhaodong, Heilongjiang, China	KX147496
		*P. pubescens* L.	PHZ2005	Baiquan, Qiqiha'er, Heilongjiang, China	KX147497
		*P. pubescens* L.	PHZ2006	Aihui, Heihe, Heilongjiang, China	KX147498
		*P. pubescens* L.	PHZ2007	Nong'an, Changchun, Jilin, China	KX147499
		*P. pubescens* L.	PHZ2008	Nong'an, Changchun, Jilin, China	KX147500
		*P. pubescens* L.	PHZ2009	Tonghua, Changchun, Jilin, China	KX147501
		*P. angustiphysa* Waterf.	–	GenBank	AY665879
		*P. cordata* Mill.	–	GenBank	AY665886
		*P. pruinosa* (Waterf.) M. Martinez	–	GenBank	AY665915
		*P. ignota* Britton	–	GenBank	AY665897
		*P. nicandroides Schlecht*.	–	GenBank	AY665912
		*P. patula* Mill.	–	GenBank	AY665913
	*Lanceolatae*	*P. caudella* Standl	–	GenBank	AY665891
		*P. hederaefolia* A. Gray	–	GenBank	AY665894
		*P. hederaefolia* var. *puberula* A. Gray	–	GenBank	AY665874
		*P. heterophylla* Nees	–	GenBank	AY665907
		*P. lanceolata* Michx.	–	GenBank	AY665899
		*P. longifolia* Nutt.	–	GenBank	AY665901
		*P. longifolia* Nutt.	–	GenBank	AY665902
		*P. peruviana* L.	–	GenBank	AY665914
		*P. peruviana* L.	–	GenBank	DQ314161
		*P. pumila* Nutt.	–	GenBank	AY665909
		*P. sordida* Fernald	–	GenBank	AY665869
		*P. virginiana* Mill.	–	GenBank	AY665911
		*P. virginiana* Mill.	–	GenBank	AY665910
	*Rydbergae*	*P. minimaculata* Waterf.	–	GenBank	AY665905
		*P. minimaculata* Waterf.	–	GenBank	AY665906
	*Viscosae*	*P. angustifolia* Nutt.	–	GenBank	AY665878
		*P. cinerascens* A. S.	–	GenBank	AY665884
		*P. cinerascens* A. S.	–	GenBank	AY665885
		*P. mollis* Nutt.	–	GenBank	AY665908
		*P. viscosa* L.	–	GenBank	AY665870
	Unknown	*P. minima* L.	PHZ3001	Tangshan, Hebei, China	KX147502
		*P. minima* L.	PHZ3002	Pingdingshan, Henan, China	KX147503
		*P. minima* L.	PHZ3003	Heze, Shandong, China	KX147504
		*P. minima* L.	PHZ3004	Lishui, Zhejiang, China	KX147505
		*P. minima* L.	PHZ3005	Lou'An, Anhui, China	KX147506
		*P. minima* L.	–	GenBank	AY665904
		*P. lassa* Stand. and Steyerm.	–	GenBank	AY665900
		*P. arenicola* Kearney	–	GenBank	AY665881
		*P. arenicola* Kearney	–	GenBank	AY665880
*Physalis*	–	*P. alkekengi* var. *franchetii* (Mast.) Makino	PHZ4001	Nong'an, Changchun, Jilin, China	KX147507
		*P. alkekengi* var. *franchetii* (Mast.) Makino	PHZ4002	Faku, Shenyang, Liaoning, China	KX147508
		*P. alkekengi* var. *franchetii* (Mast.) Makino	PHZ4003	Donggang, Dandong, Liaoning, China	KX147509
		*P. alkekengi* var. *franchetii* (Mast.) Makino	PHZ4004	Donggang, Dandong, Liaoning, China	KX147510
		*P. alkekengi* var. *franchetii* (Mast.) Makino	PHZ4005	Zhoucheng, Jinan, Shandong, China	KX147511
		*P. alkekengi* var. *franchetii* (Mast.) Makino	PHZ4006	Zhoucheng, Jinan, Shandong, China	KX147512
		*P. alkekengi* var. *franchetii* (Mast.) Makino	–	GenBank	GQ434666
		*P. alkekengi* L.	–	GenBank	AY665849
		*P. alkekengi* L.	–	GenBank	AY665850
		*P. alkekengi* L.	–	GenBank	AF244711
*Physalodendron*	–	*P. arborescens* L.	–	GenBank	AY665867
		*P. arborescens* L.	–	GenBank	AY665866
		*P. melanocystis* Bitter	–	GenBank	AY665865
*Quincula*	–	*P. walteri* Nutt.	–	GenBank	AY665918
Unknown	–	*P. microphysa* A.	–	GenBank	AY665859
*Nicandra* (Outgroup)		*N. physalodes* (L.) Gaertn.	NHZ0001	Yiwu, Zhejiang, China	KX147513
		*N. physalodes* (L.) Gaertn.	NHZ0002	Jiujiang, Jiangxi, China	KX147514
		*N. physalodes* (L.) Gaertn.	–	GenBank	LC076488
		*N. physalodes* (L.) Gaertn.	–	GenBank	DQ314155

### DNA extraction, amplification, and sequencing

Fresh, young leaf samples from *Physalis* were randomly collected for genomic DNA isolation, as described previously (Feng et al., 2013). The ITS2 sequences were amplified using the following pair of universal primers used in previous studies (Yao et al., 2010; Feng et al., 2015): ITS-2F, 5′- ATGCGATACTTGGTGTGAAT-3′ and ITS-3R, 5′-GACGCTTCTCCAGACTACAAT-3′. The primer pair was synthesized by Shanghai Sangon Biological Engineering Technology and Service Co. Ltd. (Shanghai, China). The PCR was conducted in 25 μL volumes containing 1 × PCR Buffer [100 mM Tris–HCl, 100 mM (NH_4_)_2_SO_4_, 100 mM KCl, 1% TritonX-100, pH 8.8], 2.5 mM Mg^2+^, 0.5 μM of each primer, 0.4 mM dNTPs, 1 U Taq DNA polymerase (TaKaRa Bio., Kyoto, Japan), and 50 ng genomic DNA template. The amplification was performed in a Mastercycler nexus gradient (Eppendorf AG, Hamburg, Germany) with the following PCR program: 94°C for 5 min, followed by 35 cycles of 94°C for 45 s, 56°C for 45 s, 72°C for 1.5 min, and a final extension at 72°C for 10 min. The PCR products were sequenced by Shanghai Sunny Biotechnology Co. Ltd. (Shanghai, China).

### Data analysis

The original sequences were edited and assembled manually using CodonCode Aligner V3.0 (CodonCode Co., USA). All the raw sequences were annotated and trimmed using ITS2 annotation tools based on the Hidden Markov Model (HMM) (Keller et al., 2009) to remove the conserved 5.8S and 28S DNA sequences (Koetschan et al., 2012). The trimmed sequences were edited manually. Sequences that were less than 100 bp length, or sequences that had possibly been contaminated by fungi or other unnamed species (such as those with aff. in the species name) were discarded (Nilsson et al., 2012). The selected ITS2 sequences were aligned using Clustal W (Thompson et al., 2002), and then the genetic distances were calculated using MEGA 6.0 based on the Kimura 2-Parameter (K2P) model (Tamura et al., 2013). The average inter-specific distance, the minimum inter-specific distance, and average theta prime (theta prime is the mean genetic variation between different species, thus eliminating biases associated with different numbers of samples among species) were calculated to evaluate the inter-specific divergences using the K2P model (Meyer and Paulay, 2005; Meier et al., 2008; Chen et al., 2010; Gao et al., 2010a). The average intra-specific distance, coalescent depth, and theta were used to represent the intra-specific variation based on the K2P model (Meyer and Paulay, 2005; Chen et al., 2010; Gao et al., 2010a). DNA barcoding gaps were used to compare the distributions of intra- vs. inter-specific variability (Meyer and Paulay, 2005; Chen et al., 2010; Gao et al., 2010a) and Wilcoxon two-sample tests were performed as indicated previously (Kress and Erickson, 2007; Lahaye et al., 2008b; Chen et al., 2010). BLASTA1 and the nearest distance method were used to evaluate the species authentication efficacy (Gao et al., 2010a; Feng et al., 2015). In BLASTA1 method, all ITS2 regions of *Physalis* species were used as query sequences, and BLAST program (http://blast.ncbi.nlm.nih.gov/Blast.cgi) was used for searching the reference database for each query sequence. Correct identification means that the best BLAST hit of the query sequence is from the expected species; ambiguous identification means that the best BLAST hits for a query sequence are those of several species including the expected species; and incorrect identification means that the best BLAST hit is not from the expected species (Gao et al., 2010a). In the nearest distance method, correct identification means that the hit based on the smallest genetic distances is from the same species as that of the query; ambiguous identification means that several hits have the same smallest genetic distance to the query sequence; and incorrect identification means that the hit is not from the expected species (Gao et al., 2010a). The secondary structure of *Physalis* ITS2 sequences was predicted using tools from the ITS2 database website (http://its2.bioapps.biozentrum.uni-wuerzburg.de/) (Koetschan et al., 2012). TaxonGAP 2.4.1 software was used to calculate the discriminatory power of ITS2 sequences for sister species (Slabbinck et al., 2008).

A phylogenetic analysis of the collected *Physalis* species was performed using the maximum likelihood (ML) method in MEGA 6.0 (Tamura et al., 2013). Bootstrap support (BS) values for individual clades were computed by running 1000 bootstrap replicates of the data. Four samples of *Nicandra physaloides* (Table 1), a species closely related to *Physalis* species in *Solanaceae*, were used as outgroup.

## Results

### Amplification, sequencing, and characteristics of ITS2 regions

The amplification and sequence success rate of the ITS2 sequences for the collected *Physalis* samples was 100%. The lengths of the ITS2 sequences used in the analyses ranged from 210 to 218 bp, with an average of 214 bp (Supplementary Figure 1). The GenBank accession numbers are listed in Table 1. The mean GC content was 72.4% and varied from 68.3 to 75.0% (Supplementary Figure 1). Thus, the length and GC content of the ITS2 sequences for the collected *Physalis* species were relatively variable.

### Genetic divergence within and between species

The genetic divergences of all the *Physalis* species samples were estimated using MEGA 6.0. Table 2 shows the calculated results for six metrics (average inter-specific distance, the minimum inter-specific distance, theta prime, average intra-specific distance, coalescent depth, and theta). A relatively lower divergence was observed for three metrics at the intra-specific level (Table 2).

**Table 2:** **Analyses of inter-specific divergence and intra-specific variation of the ITS2 sequences in 86 samples of 45 ***Physalis*** species**.

**Measurement**	**K2P value**
All interspecific distance	0.073 ± 0.018
Theta prime	0.068 ± 0.018
The minimum interspecific distance	0.066 ± 0.017
All intraspecific distance	0.007 ± 0.003
Theta	0.007 ± 0.003
Coalescent depth	0.010 ± 0.004

### Assessment of the barcoding gap

**Figure 2:**
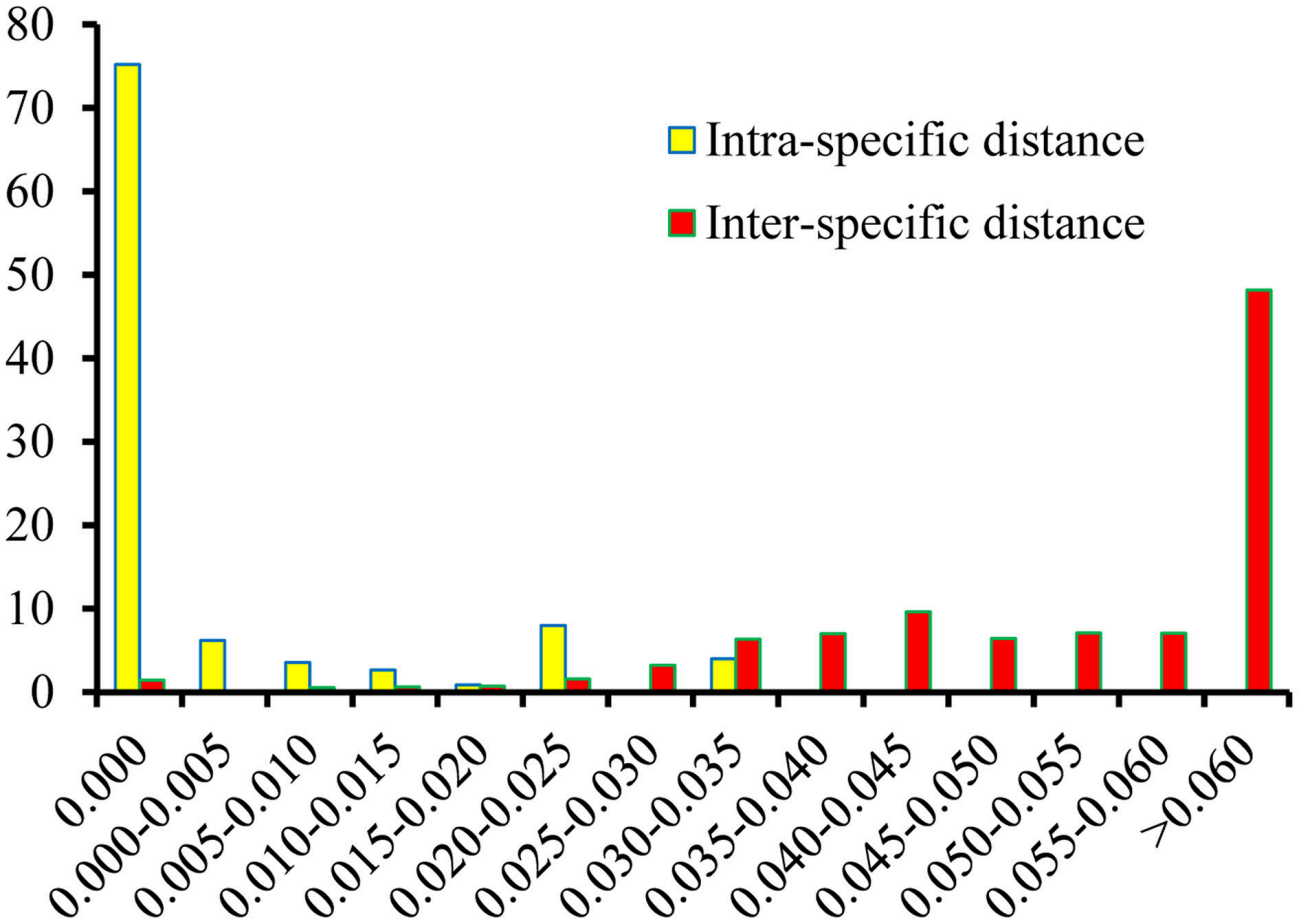
**Relative distribution of inter-specific divergence between congeneric ***Physalis*** species and intra-specific variation in the ITS2 region using K2P genetic distance**.

Based on the K2P model of intra- vs. inter-specific divergence, the distributions of genetic distance in the *Physalis* species samples were investigated at a scale of 0.005 distance units (Figure 2). The inter-specific distance ranged between 0.000 and 0.161, and equaled zero for only 1.44% of the samples. The proportion where the inter-specific genetic distance >0.035 was 85%, which will provide a useful way to authenticate different *Physalis* species. The Wilcoxon two-sample tests also indicated that there were significant differences between the inter- and intra-specific divergences (Supplementary Table 1, *P* < 0.001).

### The efficacy of ITS2 for authentication

The results showed that ITS2 possessed 82.2% identification success rates at the species level for both BLASTA1 and the nearest distance methods (Table 3). Overall, the results showed that the ITS2 region has higher identification efficiency.

**Table 3:** **Comparison of authentication efficiency for ITS2 using different methods**.

**Methods of identification**	**No. of samples**	**No. of species**	**Correct identification (%)**	**Incorrect identification (%)**	**Ambiguous identification (%)**
BLAST1	86	45	82.2	0	17.8
Distance	86	45	82.2	0	17.8

### The discriminatory power of ITS2 sequences for sister species

**Figure 3:**
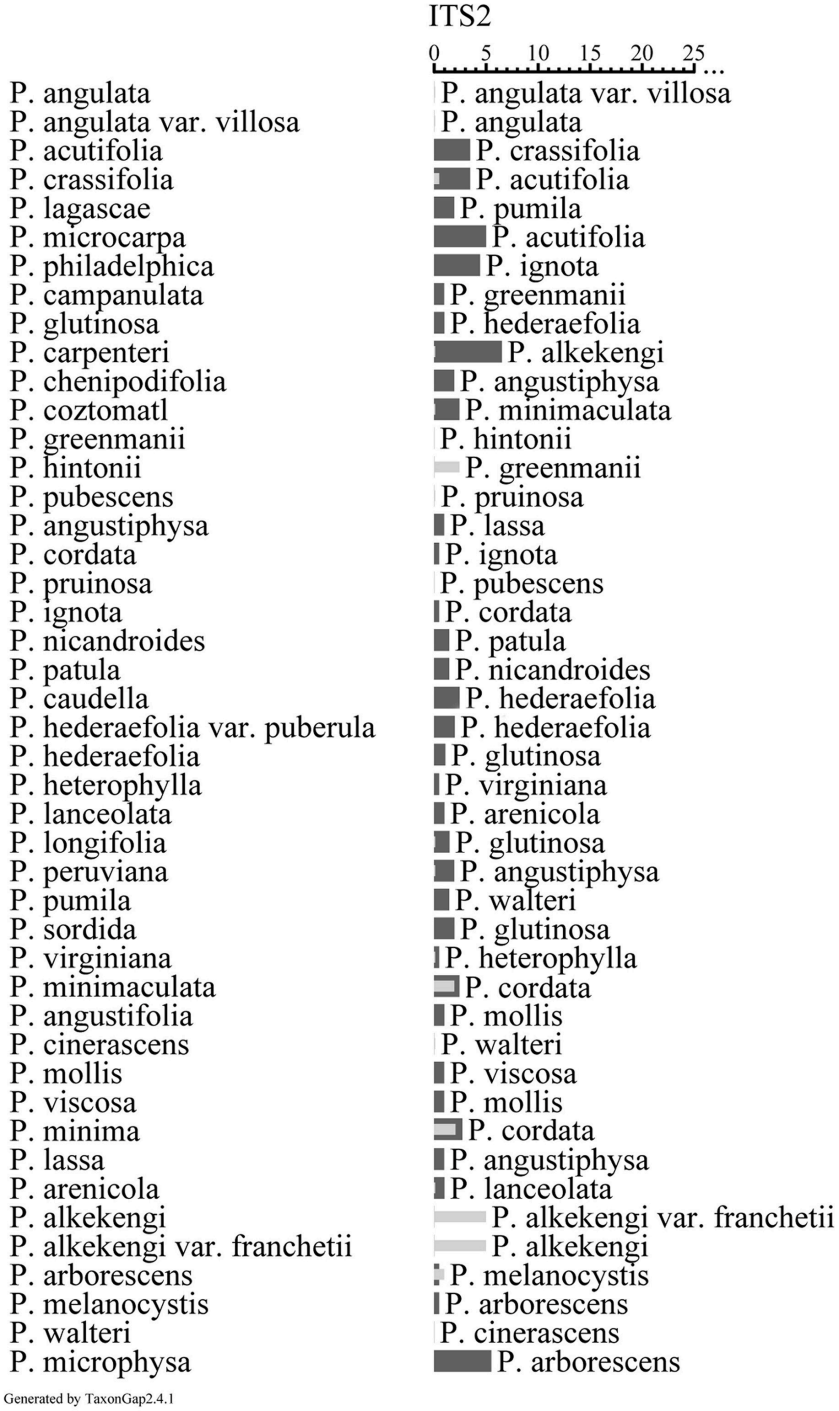
**The heterogeneity and separability for individual taxa of ITS2 based on 45 ***Physalis*** species by TaxonGap**. The left side shows the complete list of *Physalis* species used in this study. The right side depicts the within species heterogeneity (presented as light gray horizontal bar) and between-species separability (presented as dark gray horizontal bar) values with different OTUs as matrix rows for ITS2. The names of the closest relatives (the taxon with the smallest separability) are listed at the right side of the dark gray bar.

TaxonGap 2.4.1 software was used to evaluate the discriminatory power of ITS2 sequences between the collected samples (Figure 3). Over 76% of the sequences collected in this study had an inter-specific diversity that was larger than the intra-specific diversity, which indicated that the ITS2 sequences had relatively clear species boundaries. However, there were exceptions: 17.8% of the species had identical sequences with their sister-species for *P. angulate* vs. *P. angulata* var. *villosa, P. greenmanii* vs. *P. hintonii, P. pubescens* vs. *P. pruinosa*, and *P. alkekengi* vs. *P. alkekengi* var. *franchetii* (dark gray bar, Figure 3).

### Secondary structures of ITS2 regions

Besides the divergence of primary sequences of ITS2, we also focused on the use of the secondary structures of ITS2 for species identification. The secondary structures of ITS2 for collected *Physalis* species were predicted and shown in Supplementary Figure 2. All of the secondary structures of ITS2 in these species have four similar helices: Helix I, II, III, and IV (Supplementary Figure 2). However, the secondary structures of ITS2 among most *Physalis* species were variable on four helices in term loop number, size, position, and degree of angles from the center of the spiral arm. For example, the secondary structure of *P. greenmanii* was different from that of its sister-specie *P. hintonii* (Supplementary Figure 2). Similar satisfactory result was also obtained for *P. alkekengi* and *P. alkekengi* var. *franchetii*. Thus, the secondary structure of ITS2 provided another method for *Physalis* species identification. However, identical secondary structures were found in *P. angulate* vs. *P. angulata* var. *villosa*, and *P. pubescens* vs. *P. pruinosa* (Supplementary Figure 2).

### Phylogenetic analysis

**Figure 4:**
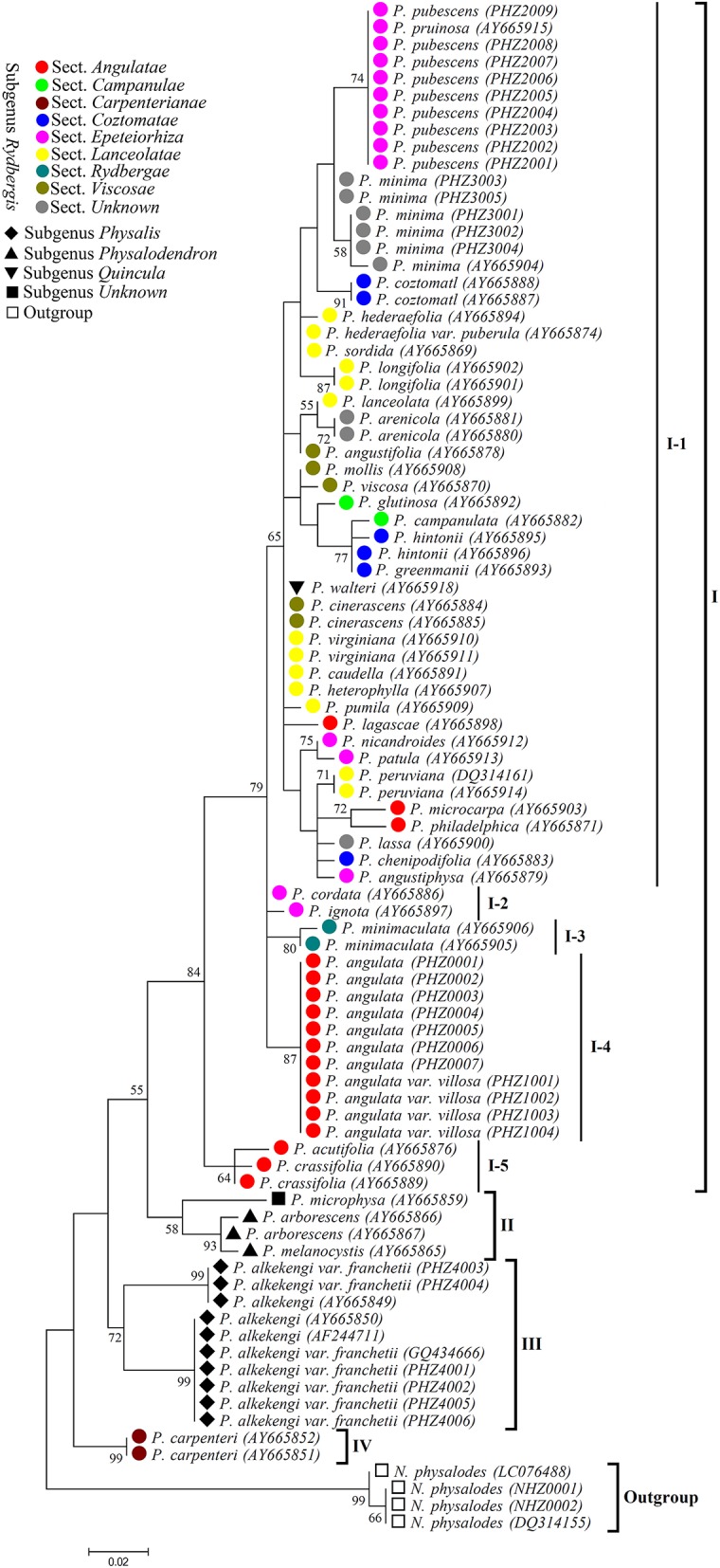
**Maximum likelihood (ML) tree based on ITS2 sequences for ***Physalis*** species**. Numbers above branches indicate bootstrap support (BS ≥ 50) values.

According to the *Physalis* species morphological classification reported in previous studies (Axelius, 1996; Maggie, 2005), all the *Physalis* species collected in this study belonged to four subgenera (*Rydbergis, Physalis, Physalodendron*, and *Quincula*), in addition to one species without grouping (we grouped it in subgenus Unknown in this study). In subgenus *Rydbergis*, most species were grouped into eight sections (sect.): *Angulatae, Campanulae, Carpenterianae, Coztomatae, Epeteiorhiza, Lanceolatae, Rydbergae*, and *Viscosae*. In addition, three species: *P. minima, P. lassa*, and *P. arenicola*, did not have a grouping based on morphology in subgenus *Rydbergis* (we grouped these species in section Unknown in this study). In this study, a dendrogram constructed by the ML method based on ITS2 sequences grouped all the *Physalis* species into four main clusters (Figure 4).

Group I was the most complex, with 38 species, and was further divided into five subgroups. In addition to the species from sect. *Carpenterianae*, all species from the other eight sections of subgenus *Rydbergis* were included in Group I. *P. walteri*, a species from subgenus *Quincula* was also grouped into Group I. Subgroup I-1 contained 31 species: five from sect. *Epeteiorhiza*, four from sect. *Coztomatae*, ten from sect. *Lanceolatae*, four from sect. *Viscosae*, three form sect. *Angulatae*, two from sect. *Campanulae*, three from sect. unknown of subgenus *Rydbergis*, and the species from subgenus *Quincula*. Subgroup I-2 included two species (*P. cordata* and *P. ignota*) from sect. *Epeteiorhiza*. Subgroup I-3 contained the species (*P. minimaculata*) from subgenus *Rydbergae*. Subgroup I-4 contained two species (*P. angulate* and *P. angulate* var. *villosa*) form sect. *Angulatae*. *P. acutifolia* and *P. crassifolia* from sect. *Angulatae* were grouped into Subgroup I-5.

Group II contained three species, including all species (*P. arborescens* and *P. melanocystis*) from subgenus *Physalodendron* and the species *P. microphysa* from subgenus Unknown. *P. alkekengi* and *P. alkekengi* var. *franchetii* from subgenus. *Physalis* constituted a separate group III. *P. carpenteri* from sect. *Carpenterianae* of subgenus. *Rydbergis* was distant from any other *Physalis* species, and was assigned into group IV.

## Discussion

*Physalis* species are important medicinal and edible plants that have a significant, economic value. DNA barcoding, using the ribosomal DNA ITS2 region as a tag to identify species, has recently attracted much attention (Chen et al., 2010). Compared with other candidate DNA barcodes, such as *psbA-trnH, matK, rbcL*, and ITS, ITS2 possesses many advantages, including good universality, small intraspecific variation, but high interspecific divergence, and a small fragment length (~200 bp; Chen et al., 2010; Yao et al., 2010). To our knowledge, this is the first time that the ITS2 regions have been used to identify *Physalis* species in such a large sample size, an endeavor which has expanded the application of the ITS2 region to the medicinal plant field.

As in some previous studies (Gao et al., 2010a; Liu et al., 2012; Feng et al., 2015), sufficient variation was found in the ITS2 region among *Physalis* species to allow determination of genetic divergence, and the ITS2 region also demonstrated a higher successful discrimination capability (compared to 82.2% identification success rates for both BLASTA1 and the nearest genetic distance methods). For example, *P. angulata* and *P. minima* have extremely similar morphological traits, rendering their differentiation very difficult and sometimes impossible (Figure 1), but they could be accurately discriminated based on their ITS2 regions. In addition, because of sufficient variation in the ITS2 secondary structures, some studies suggested that the secondary structure of ITS2 might be considered as a molecular morphological characteristic (Yao et al., 2010). In this study, we found that the secondary structures of ITS2 perform well in identifying *Physalis* species (Supplementary Figure 2). For example, some species (*P. greenmanii* vs. *P. hintonii*, and *P. alkekengi* vs. *P. alkekengi* var. *franchetii*) from the ambiguous identification cases by the BLAST 1 or nearest distance method could be identified by their ITS2 secondary structures (Supplementary Figure 2).

The ITS2 region cannot solve all the species identification problems in *Physalis*. For example, *P. pubescens* vs. *P. pruinosa, P. angulate* vs. *P. angulata* var. *villosa* were found to have identical ITS2 sequences and the same secondary structures,. Therefore, it might be worthwhile using other DNA barcodes as complementary factors for discriminating these species. Certainly, it should be noted that the taxonomic assignment of sequences from GenBank might not be accurate due to the similar morphological characteristics of some *Physalis* species (for example: *P. pubescens* and *P. pruinosa*). Hence, if these factors were taken into account, estimations of in-species discrimination might be lower for *Physalis*.

Some studies have suggested that although DNA barcoding aims to provide an efficient method for species-level identification, it may also contribute to taxonomic and biodiversity research (Hajibabaei et al., 2007; Wang et al., 2010; Wong et al., 2011; Feng et al., 2015). The ITS2 region could provide taxonomic signatures in systematic evolution (Coleman, 2003; Schultz et al., 2005; Liu et al., 2012; Feng et al., 2015). In our study, ITS2 could be used to barcode *Physalis* and to serve as a phylogenetic marker for *Physalis* taxonomy. As in previous studies (Mione et al., 1994; Olmstead et al., 1999; Maggie, 2005), the dendrogram constructed with ITS2 data using the ML method indicated that the genus *Physalis* was paraphyletic. Maggie (2005) showed that subgenus *Rydbergis* was morphologically homogeneous and we obtained similar results that showed that most collected species of subgenus *Rydbergis* (except *P. carpenteri* from sect. *Carpenterianae*) were grouped into group I. The species in subgenera *Physalodendron*, and *Physalis* were all morphologically atypical, either having multiple flowers per node, corollas which are lobed or odd colors, or unusual fruiting calyx morphology (Martinez, 1999; Maggie, 2005). In our study, these species were distant from the species in subgenus *Rydbergis*, and were grouped into groups II and III, respectively (Figure 4). *P. walteri* from subgenus *Quincula* was included within group I (I-1) together with species from sect. *Viscosae* (subgenus *Rydbergis*). In fact, *P. walteri* was strongly supported as sister species to *P. viscosa* and *P. angustifolia* (Waterfall, 1967; Maggie, 2005). In addition, we found that *P. microphysa* from subgenus Unknown, was an unplaced species in a previous study (Maggie, 2005). However, we were able to group it into group II together with species in subgenus *Physalodendron* with weak support (*BS* = 58). Thus, it appears that more sampling and more up-to-date phylogenetic methods are required to understand the taxonomy of *P. microphysa*.

Although most of the species in subgenus *Rydbergis* were grouped together within group I, some sections of subgenus *Rydbergis* were probably polyphyletic, such as *Epeteiorhiza* (I–1, I–2), and *Angulatae* (I–1, I–4, I–5) (Figure 4). In addition, the species form sect. *Lanceolatae* were Clustered together with the species from other sections (such as *Viscosae, Angulatae*, and *Epeteiorhiza*). Similar results have been reported by Maggie (2005). As for sect. *Carpenterianae, P. carpenteri* along with other species from other sections of subgenus *Rydbergis*, formed group IV with strong support (*BS* = 99) as previously reported (Maggie, 2005). Our results largely concurred with the view of the previous study that *P. carpenteri, P. alkekengi, P. microphysa*, and subgenus *Physalodendron* should be recognized as four small genera (Maggie, 2005).

Some studies have concluded that DNA barcoding sequences do not usually have sufficient phylogenetic signals to resolve evolutionary relationships (Hajibabaei et al., 2006). In addition, multiple copies of ITS2 may suggest that the sequences obtained through PCR are not stable and representative and this might result in misleading phylogenetic inferences (Queiroz Cde et al., 2011). However, after comparison with previous studies, our results have demonstrated that ITS2 is a useful DNA barcode that could be used to identify *Physalis* species, and build relatively reliable molecular phylogenies for the genus *Physalis*.

## Author contributions

Conceived and designed the study: SF, HW. Collected plant samples: SF, HW, YS, JL, and QY. Performed the experiments: SF, MJ, KJ, and CS. Analyzed the data: SF, MJ, KJ. Wrote the manuscript: SF, HW.

## Funding

This study was supported in part by the National Natural Science Foundation of China (31470407), the Zhejiang Provincial Public Welfare Technology Applied Research Foundation of China (2014C32090), the Hangzhou Scientific and Technological Program (20150932H04), and the Hangzhou Scientific and Technological Program (20150932H03).

### Conflict of interest statement

The authors declare that the research was conducted in the absence of any commercial or financial relationships that could be construed as a potential conflict of interest.

## Supplementary material

Supplementary Figure 1 **Distribution of sequence lengths and GC content of the ITS2 sequences from all ***Physalis*** species**.

Supplementary Figure 2 **The secondary structure of ITS2 in different ***Physalis*** species**. (1) *P. angulata* (PHZ0001), (2) *P. angulata* var. *villosa* (PHZ1001), (3) *P. acutifolia* (AY665876), (4) *P. crassifolia* (AY665890), (5) *P. lagascae* (AY665898), (6) *P. microcarpa* (AY665903), (7) *P. philadelphica* (AY665871), (8) *P. campanulata* (AY665882), (9) *P. glutinosa* (AY665892), (10) *P. carpenteri* (AY665852), (11) *P. chenipodifolia* (AY665883), (12) *P. coztomatl* (AY665888), (13) *P. greenmanii* (AY665893), (14) *P. hintonii* (AY665895), (15) *P. pubescens* (PHZ2001), (16) *P. angustiphysa* (AY665879), (17) *P. cordata* (AY665886), (18) *P. pruinosa* (AY665915), (19) *P. ignota* (AY665897), (20) *P. nicandroides* (AY665912), (21) *P. patula* (AY665913), (22) *P. caudella* (AY665891), (23) *P. hederaefolia* (AY665894), (24) *P. hederaefolia* var. *puberula* (AY665874), (25) *P. heterophylla* (AY665907), (26) *P. lanceolata* (AY665899), (27) *P. longifolia* (AY665901), (28) *P. peruviana* (AY665914), (29) *P. pumila* (AY665909), (30) *P. sordida* (AY665869), (31) *P. virginiana* (AY665910), (32) *P. minimaculata* (AY665905), (33) *P. angustifolia* (AY665878), (34) *P. cinerascens* (AY665884), (35) *P. mollis* (AY665908), (36) *P. viscosa* (AY665870), (37) *P. minima* (AY665904), (38) *P. lassa* (AY665900), (39) *P. arenicola* (AY665880), (40) *P. alkekengi* (AY665849), (41) *P. alkekengi* var. *franchetii* (PHZ4001), (42) *P. arborescens* (AY665866), (43) *P. melanocystis* (AY665865), (44) *P. walteri* (AY665918), (45) *P. microphysa* (AY665859).

Supplementary Table 1 **Wilcoxon two-sample tests for distribution of intra- vs. inter-specific divergences**.
