# Supplementary figures and images for: Application of the Ribosomal DNA ITS2 Region of Physalis (Solanaceae): DNA Barcoding and Phylogenetic Study
Source: Front Plant Sci. 2016 Jul 19;7:1047. doi: 10.3389/fpls.2016.01047 (PMC4949264; doi:10.3389/fpls.2016.01047)

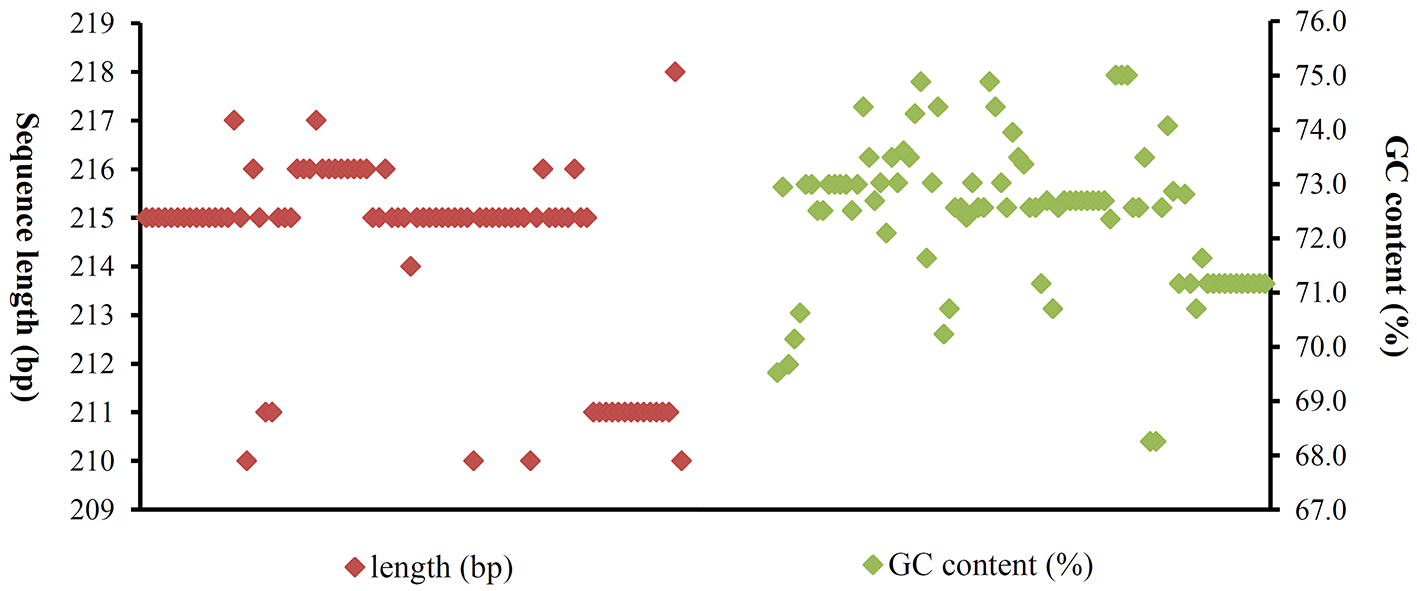

Supplement: Supplementary Figure 1 — Distribution of sequence lengths and GC content of the ITS2 sequences from all Physalis species. [file Image1.TIF]

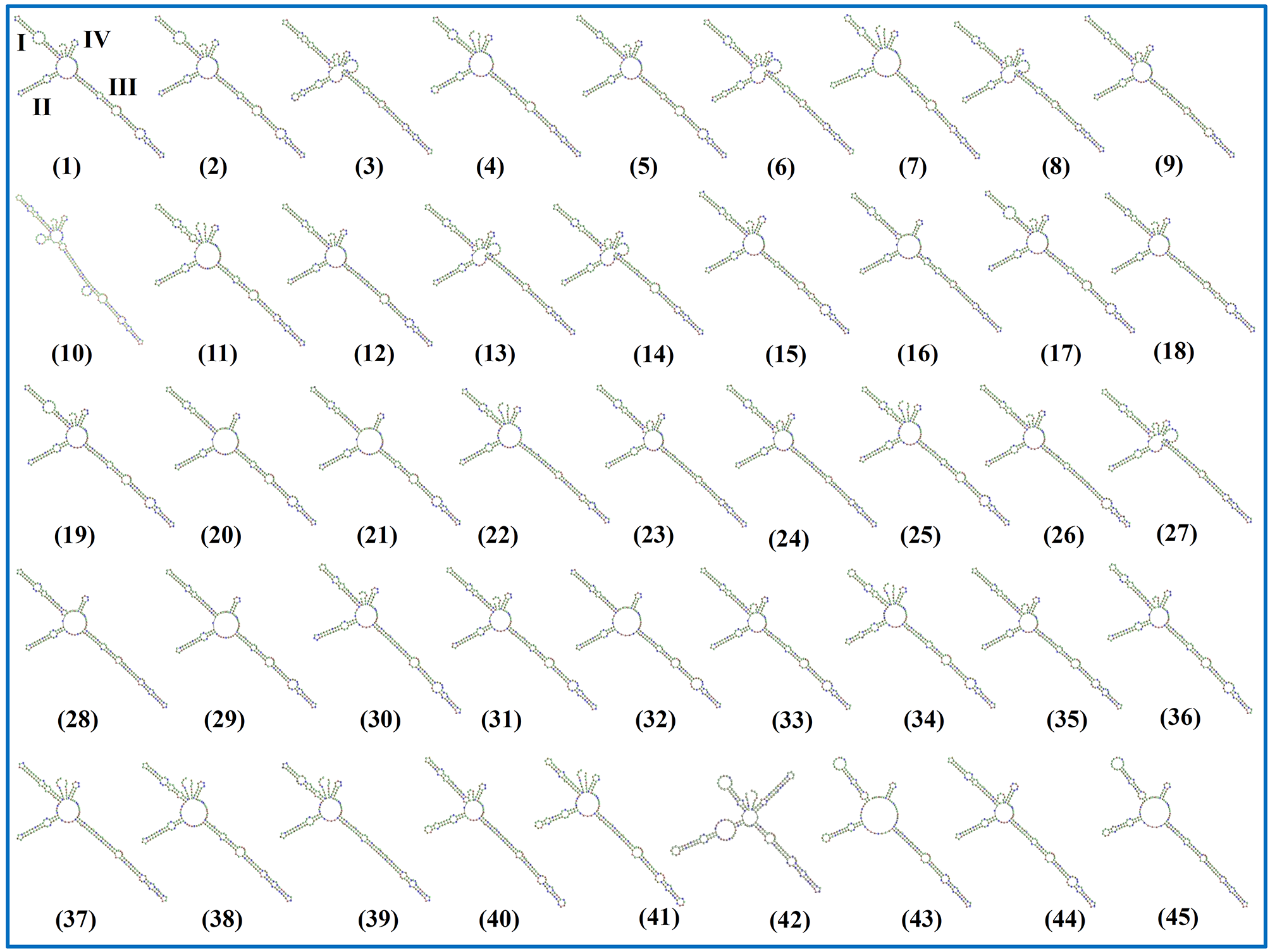

Supplement: Supplementary Figure 2 — The secondary structure of ITS2 in different Physalis species. (1) P. angulata (PHZ0001), (2) P. angulata var. villosa (PHZ1001), (3) P. acutifolia (AY665876), (4) P. crassifolia (AY665890), (5) P. lagascae (AY665898), (6) P. microcarpa (AY665903), (7) P. philadelphica (AY665871), (8) P. campanulata (AY665882), (9) P. glutinosa (AY665892), (10) P. carpenteri (AY665852), (11) P. chenipodifolia (AY665883), (12) P. coztomatl (AY665888), (13) P. greenmanii (AY665893), (14) P. hintonii (AY665895), (15) P. pubescens (PHZ2001), (16) P. angustiphysa (AY665879), (17) P. cordata (AY665886), (18) P. pruinosa (AY665915), (19) P. ignota (AY665897), (20) P. nicandroides (AY665912), (21) P. patula (AY665913), (22) P. caudella (AY665891), (23) P. hederaefolia (AY665894), (24) P. hederaefolia var. puberula (AY665874), (25) P. heterophylla (AY665907), (26) P. lanceolata (AY665899), (27) P. longifolia (AY665901), (28) P. peruviana (AY665914), (29) P. pumila (AY665909), (30) P. sordida (AY665869), (31) P. virginiana (AY665910), (32) P. minimaculata (AY665905), (33) P. angustifolia (AY665878), (34) P. cinerascens (AY665884), (35) P. mollis (AY665908), (36) P. viscosa (AY665870), (37) P. minima (AY665904), (38) P. lassa (AY665900), (39) P. arenicola (AY665880), (40) P. alkekengi (AY665849), (41) P. alkekengi var. franchetii (PHZ4001), (42) P. arborescens (AY665866), (43) P. melanocystis (AY665865), (44) P. walteri (AY665918), (45) P. microphysa (AY665859). [file Image2.TIF]
