# Supplementary material for: Application of the Ribosomal DNA ITS2 Region of Physalis (Solanaceae): DNA Barcoding and Phylogenetic Study
Source: Front Plant Sci. 2016 Jul 19;7:1047. doi: 10.3389/fpls.2016.01047 (PMC4949264; doi:10.3389/fpls.2016.01047)
Supplement: Supplementary Table 1 — Wilcoxon two-sample tests for distribution of intra- vs. inter-specific divergences. [file Table1.DOC]

**Supplementary Material 2** Wilcoxon two-sample tests for distribution of intra- vs. inter-specific divergences

| No. of inter-specific distances | No. of intra-specific distances | Wilcoxon W | *P* value |
| --- | --- | --- | --- |
| 3491 | 2.50 | 6095283.50 | 0.000 |
